# Periacetabular osteotomy of the hip: an 8-year follow-up of 96 consecutive cases

**DOI:** 10.1007/s00402-026-06298-0

**Published:** 2026-04-17

**Authors:** Anders Enocson, Richard Wallensten, Henrik Lundblad

**Affiliations:** 1 Department of Molecular Medicine and Surgery, Karolinska Institutet, Stockholm, Sweden; 2 Department of Trauma and Orthopaedics, Karolinska University Hospital, Stockholm, Sweden

**Keywords:** Periacetabular osteotomy, Bernese osteotomy, Hip dysplasia, Joint-preserving surgery, Total hip arthroplasty

## Abstract

**Introduction:**

A periacetabular osteotomy (PAO) is a joint-preserving surgical option for treatment of acetabular dysplasia. The procedure aims to prevent, or at least delay, the development of osteoarthritis, and subsequent need for total hip arthroplasty (THA). The conversion rate to THA differs widely in the literature, but most of the studies have few patients, and the follow-up time is often short for THA as an endpoint. The aim of this study was to evaluate the long-term outcome after PAO surgery with the rate of conversion to THA as the primary outcome.

**Materials and methods:**

Patients ≥18 years that underwent a PAO operation at the Karolinska University Hospital in Stockholm, Sweden from 2006 to 2022 were included. Radiological signs of hip osteoarthritis, and the lateral center-edge angle (LCEA) was calculated on pre- and postoperative radiographs or CT-scans. The national Swedish Arthroplasty Register was used to find cases who had a secondary operation with THA.

**Results:**

The number of cases included was 96. Median age was 30 (18–46) years, and 84% (*n* = 81) were females. Median follow-up time was 99 (17–227) months (8 years). A total of 21 (22%) cases had a secondary THA. Cox regression analyses identified that age ≥ 30 years and smoking was associated with THA reoperation in both uni- (HR 2.8, CI 1.1–7.3, HR 3.7, CI 1.0–13) and multivariable (HR 4.0, CI 1.1–15, HR 7.8, CI 1.7–37) analyses. Preoperative osteoarthritis (Tönnis grade 2) was weakly associated with THA in multivariable (HR 31, CI 2.6–384) analysis. A total of 10 (10%) cases had an unexpected reoperation due to other reasons than a secondary THA, and the most common reason was nonunion (*n* = 6, 6.3%). Forty-four (43%) patients had an adverse event. The most common was a transient nerve injury.

**Conclusions:**

The PAO procedure is a suitable option in young patients with symptomatic dysplasia of the hip in order to avoid, or at least delay, hip arthroplasty.

## Background

Acetabular dysplasia is a well-known risk factor for early-onset hip osteoarthritis in younger patients [1]. A periacetabular osteotomy (PAO) is a joint-preserving surgical option for treatment of acetabular dysplasia [2]. The rationale for this surgical method is that the shallow dysplastic acetabulum causes mechanical instability and increased focal pressure on the cartilage of the hip joint, and left untreated often result in early development of osteoarthritis [3, 4]. A nowadays commonly used surgical technique for PAO was described in 1988 by Ganz [5] and is often referred to as the Bernese PAO. In this operation, the acetabulum is reoriented to increase the anterolateral coverage of the femoral head and to achieve medialization of acetabulum, thereby increasing the cartilage joint contact surface and mechanical stability of the joint [5]. The procedure aims to prevent, or at least delay, the development of osteoarthritis, and subsequent need for total hip arthroplasty (THA) [6]. The PAO procedure has been associated with relatively high patient satisfaction in general, but also an inversed correlation between severity of dysplasia and patient satisfaction, with more dysplastic hips having lower rates of postoperative satisfaction [7]. Furthermore, preoperative patient evaluation and selection seems to be crucial as postoperative functional recovery varies widely [8, 9]. Postoperative outcome after surgical procedures can be measured in several ways, and in the case of PAO the rate of conversion to THA is of major interest. In a recently published systematic review and meta-analysis with a mean follow-up of 54 months, it was reported that 6% of the patients underwent a secondary THA after a PAO [6]. However, most of the included studies had few patients, and the follow-up time was short if THA surgery is to be used as an endpoint. Several authors have reported favorable functional outcomes in patient reported measures (PROMs) after PAO surgery [10–12]. The aim of this study was to evaluate the long-term outcome after Bernese PAO surgery with the rate of conversion to THA as the primary outcome. Secondary outcomes included radiological findings, other reoperations and adverse events.

## Patients and methods

All patients ≥18 years that underwent a PAO at the Karolinska University Hospital in Stockholm, Sweden from 2006 to 2022 were included in this retrospective cohort study. Patients were identified through the local surgical database and all medical records including radiographs were manually reviewed. A letter with information about the study with the option to opt-out was sent to all patients.

Collected demographic variables included patient age, gender, diagnosis and indication for surgery. Radiological signs of hip osteoarthritis on preoperative radiographs or CT-scans were classified according to Tönnis [13]. The lateral center-edge angle (LCEA) was calculated on pre- and postoperative radiographs or CT-scans [14] and classified as; normal (> 25°), borderline (20–25°) or dysplastic (< 20°) [15]. The anterior and posterior wall index (AWI, PWI) was calculated and classified according to Siebenrock et al. [16], as well as the acetabular retroversion index which was calculated and classified as normal or increased (> 0.3) [17]. The indication for the surgery was symptoms from the hip (pain) and radiographs indicating dysplastic anatomy of the hip.

To find cases who had a secondary operation with a total hip arthroplasty, cross-referencing was performed with the national Swedish Arthroplasty Register (SAR). The SAR started in 1979 and collects detailed data on all primary and secondary hip arthroplasties performed in Sweden in patients with a valid Swedish personal identification number. The completeness in SAR for primary total hip arthroplasty procedures has been > 98% over the last 10 years (www.slr.registercentrum.se).

Other follow-op variables included any other reoperation, including cause and type, any adverse event not requiring surgical treatment (nerve injury, pneumonia, pulmonary embolism, deep venous thrombosis, urinary tract infection, sepsis, kidney failure, superficial wound infection). In addition, mortality was recorded. All patients were followed for a minimum of 2 years after the surgery.

All surgeries were performed in a supine position under general anesthesia with intraoperative fluoroscopy. The surgical incision used was a shortened ileo-inguinal, and the fixation of the osteotomy was normally done with 3 fully threaded 3.5 mm cortical screws. Perioperative intravenous antibiotic prophylaxis was given to all patients, as well as postoperative low-molecular-weight heparin for prevention of blood clots. Postoperatively, patients were instructed to partial weight-bear the operated side using crutches for the first 6–8 weeks.

The study was approved by the Swedish Ethical Review Authority with reference number: 2023-067811-01.

### Statistical methods

Patients with bilateral operations were analyzed as two separate cases. Numerical data was presented as median (range). Categorical data was presented as frequency with percent distribution. Nominal variables were tested with the Fisher’s exact test. The Wilcoxon signed rank test or the Mann-Whitney U test were used for comparison of scale variables. All tests were two-sided. Cox regression analysis was performed to evaluate factors associated with THA reoperation. Age (< 30 or ≥ 30 years (median age)), smoker (yes or no), preoperative Tönnis grade (0–3), preoperative LCEA (dysplasic, borderline or normal), postoperative AWI (deficient, normal or excessive), postoperative PWI (deficient, normal or excessive), postoperative ARI (normal or increased) was included in the analyses. First, crude association for each variable was tested in univariable models. Second, a multivariable model was used to study the adjusted associations. The associations were presented as hazard ratios (HRs) with 95% confidence intervals (CIs). The follow-up time was defined as the time from the date of PAO surgery to the date of THA surgery, death or December 31, 2024. The results were considered significant at *p* < 0.05. The statistical software used was IBM SPSS Statistics, Version 31 for Windows (SPSS Inc., Chicago, Illinois).

## Results

A total of 84 patients were identified. Two patients had emigrated abroad, and two patients replied with an opt-out of the study, leaving 80 patients to be included. Of those, 16 patients were operated in both hips, all at different occasions. Thus, the total number of cases/hips included in the analysis was 96. Median age at the operation was 30 (18–46) years, and 84% (*n* = 81) were females (Table 1). Median follow-up time was 99 (17–227) months (8 years). All patients were alive at the closure of the study (December 31, 2024) Fig. 1.

**Table 1:** Patient characteristics including comparisons between patients that underwent subsequent THA surgery (THA), and those who did not (No THA)

Variable	All cases (*n* = 96)	THA (*n* = 21)	No THA (*n* = 75)	*p*-value
Age; Median (range)	30 (18–46)	39 (18–44)	28 (18–46)	0.02
Female gender; n= (%)	81 (84)	20 (95)	61 (81)	0.2
Operated hip left side; n= (%)	44 (46)	9 (43)	35 (47)	0.8
Smoker; n= (%)	4 (4.2)	3 (14)	1 (1.3)	0.03
Diagnosis; n= (%) Primary dysplasia Secondary dysplasia due to Perthes	94 (98) 2 (2.1)	21 (100) 0	73 (97) 2 (2.7)	1.0

*THA* total hip arthroplasty

**Fig. 1:**
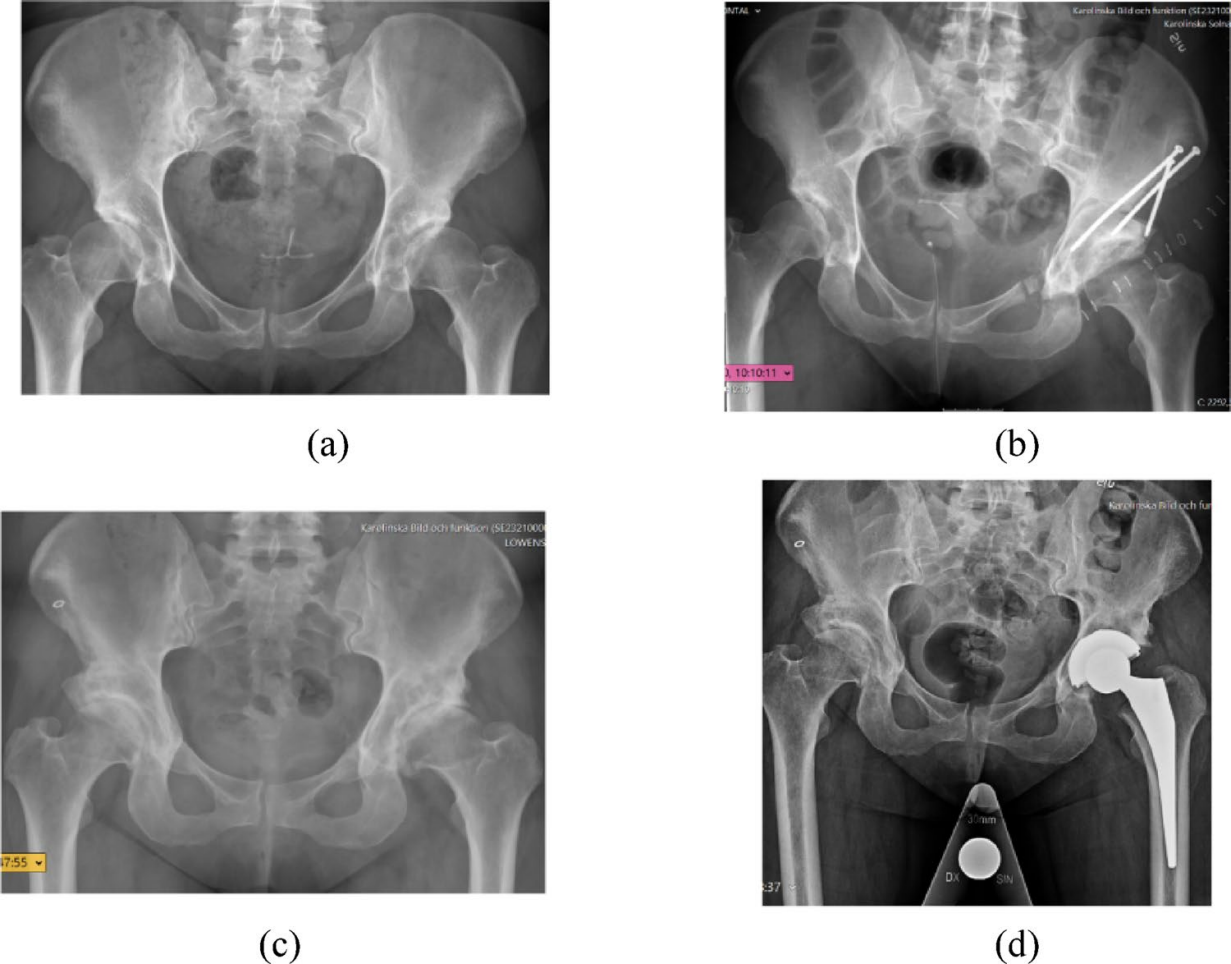
**a**-**d**. A female with bilateral dysplasia (**a**), who underwent a PAO operation in the left hip (**b**). She later developed osteoarthritis in the left hip (**c**), and was operated with an uncemented THA 8 years after the PAO operation (**d**)

### Radiographic analyses

For one patient, preoperative radiographs could not be retrieved. In the remaining cases, 77 (81%) had Tönnis grade 0, 16 (17%) cases had grade 1 and 2 cases (2%) had grade 2 radiological signs of hip osteoarthritis. For the calculation of acetabular radiological parameters (LCEA, AWI, PWI and ARI), two patients had radiographs insufficient for calculation of preoperative AWI, PWI and ARI and one for preoperative LCEA. Preoperative, 75 (78%) cases had a dysplastic (< 20°), 14 (15%) cases had a borderline (20–25°), 6 (6.3%) cases had a normal (> 25°) LCEA. The median LCEA, AWI and ARI increased from pre- to postoperative, and the median PWI decreased (Table 2).

**Table 2:** Pre- and postoperative acetabular radiological parameters; Median (range)

Variable	Preoperative	Postoperative	*p*-value
LCEA (°)	13 (0–29)	36 (14–58)	< 0.001
AWI	0.26 (-0.08-0.67)	0.4 (-0.16-0.99)	< 0.001
PWI	0.73 (0.19–1.17)	0.63 (0.11–1.13)	< 0.001
ARI	0.04 (0-0.43)	0.17 (0-0.73)	< 0.001

*LCEA*  lateral center-edge angle,* AWI *anterior wall index,* PWI* posterior wall index,* ARI *acetabular retroversion index

### Secondary operation with THA

**Fig. 2:**
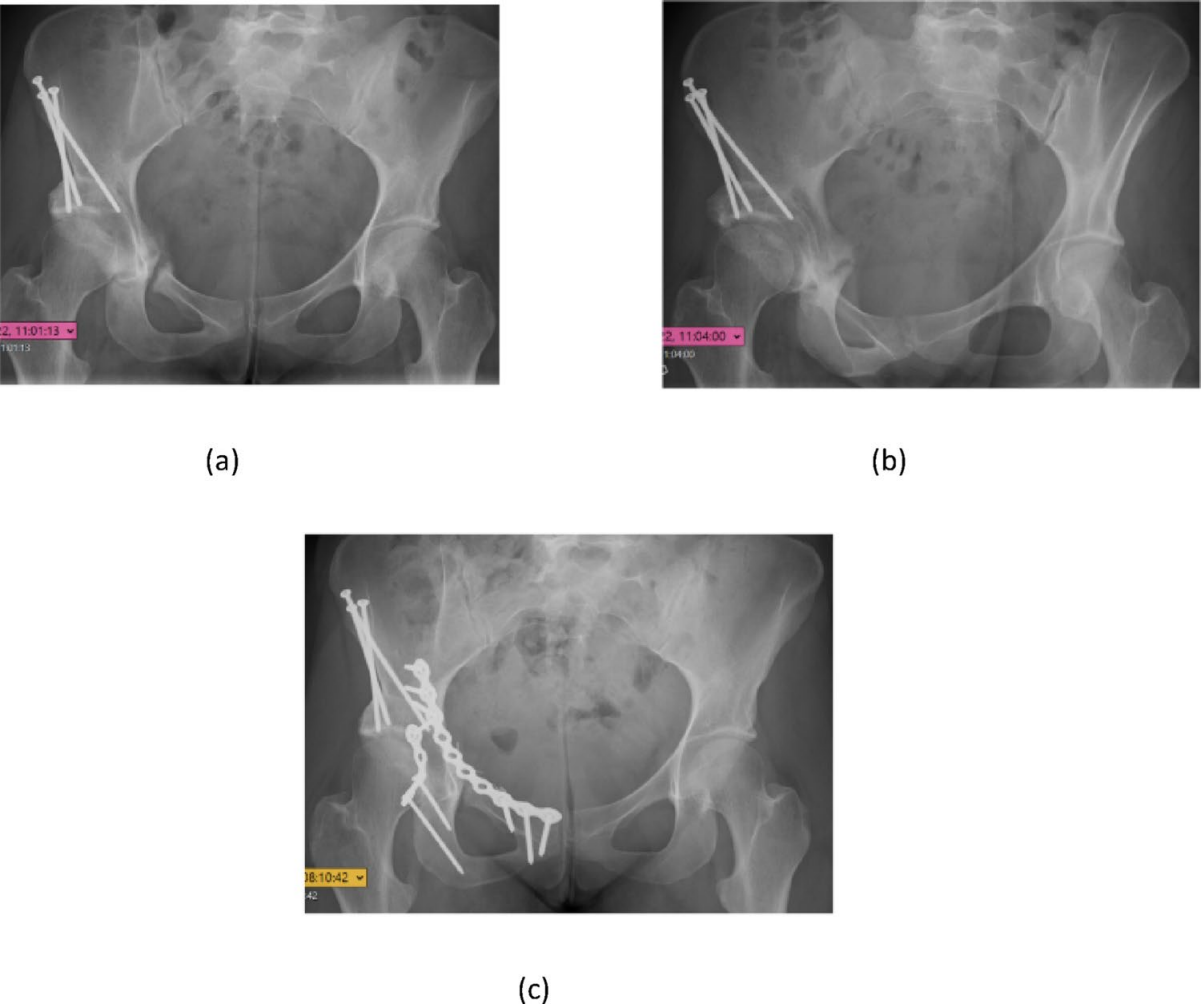
**a**-**c**. A female with nonunion 16 months after a PAO operation (**a + b)**. She was reoperated with anterior and posterior plating and went on to healing after 7 months (**c**)

After cross-referencing with the SAR, a total of 21 (22%) cases were identified that had a secondary operation with a THA. The median age of these patients at the time of the THA surgery was 43 (28–54) years, and 20 were females (Table 1). An uncemented arthroplasty was the most commonly used type of THA (*n* = 17), followed by a reverse hybrid type (cemented cup and uncemented stem) (*n* = 3) or a hybrid type (uncemented cup and cemented stem) (*n* = 1) (Fig. 2). The median time to the THA operation was 57 (17–214) months. Cox regression analyses identified that age ≥ 30 years and smoking was associated with THA reoperation in both uni- (HR 2.8, CI 1.1–7.3, HR 3.7, CI 1.0–13) and multivariable (HR 4.0, CI 1.1–15, HR 7.8, CI 1.7–37) analyses. Preoperative osteoarthritis (Tönnis grade 2) was associated with THA reoperation in multivariable (HR 31, CI 2.6–384) analysis (Table 3).

**Table 3:** Cox regression to evaluate factors associated with risk for reoperation with THA

Variable	*n*=	Reoperation	Univariable	Multivariable
		*n*= (%)	HR (95%CI)	HR (95%CI)
*Age*				
< 30 years ≥ 30 years	46 50	6 (13) 15 (30)	1 (reference) 2.8 (1.1–7.3)	1 (reference) 4.0 (1.1–15)
*Smoker*				
No Yes	92 4	18 (20) 3 (75)	1 (reference) 3.7 (1.0–13)	1 (reference) 7.8 (1.7–37)
*Preoperative Tönnis* ^1^				
0 1 2	77 16 2	14 (18) 5 (31) 1 (50)	1 (reference) 1.7 (0.6–4.8) 6.0 (0.7–48)	1 (reference) 1.4 (0.5–4.3) 31 (2.6–384)
*Preoperative LCEA* ^1^				
< 20° Dysplastic 20–25° Borderline > 25° Normal	75 14 6	18 (24) 2 (14) 0 (0)	1 (reference) 0.8 (0.2–3.3) 0	1 (reference) 0.6 (0.1–3.2) 0
*Postoperative AWI*				
< 0.3 Deficient 0.3–0.51 Normal > 0.51 Excessive	27 40 29	5 (19) 10 (25) 6 (21)	1 (reference) 1.6 (0.5–4.6) 1.8 (0.5–6.1)	1 (reference) 1.3 (0.4–4.2) 2.1 (0.5–9.2)
*Postoperative PWI*				
< 0.81 Deficient 0.81–1.14 Normal > 1.14 Excessive	77 19 0	17 (22) 4 (21) 0	1 (reference) 0.9 (0.3–2.6) NA	1 (reference) 1.2 (0.4-4.0) NA
*Postoperative ARI*				
0-0.3 Normal > 0.3 Increased	65 31	15 (23) 6 (19)	1 (reference) 1.0 (0.4–2.6)	1 (reference) 0.7 (0.2–2.3)

*THA*  total hip arthroplasty,* HR*  hazard ratio,* CI * confidence interval,* LCEA*  lateral center-edge angle,* AWI*  anterior wall index,* PWI *posterior wall index,* ARI*  acetabular retroversion index

^1^ one missing case

### Reoperations

A total of 10 (10%) cases had an unexpected reoperation due to other reasons than a secondary THA. The most common reason for reoperation was nonunion (*n* = 6, 6.3%) followed by infection (*n* = 2, 2.1%), postoperative bleeding (*n* = 1, 1.0%) and nerve exploration (*n* = 1, 1.0%). One of the nonunion patients was a smoker. Three patients underwent multiple reoperations: one patient with nonunion was reoperated three times (plate fixation x 2, later extraction of implants), another patient with nonunion was also reoperated three times (plate fixation, replacement of screw penetrating the joint, later extraction of implants) and one patient with nonunion was reoperated twice (extraction of screws, plate fixation) (Fig. 2). The median time to the first reoperation due to nonunion was 13 (6–21) months. None of these 3 patients were converted to THA. In addition, 39 patients (41%) were reoperated with extraction of screws. The median time to reoperation with extraction of screws was 17 (2-113) months.

### Other adverse events

Forty-four (43%) patients had an adverse event. The most common was an injury to the lateral femoral cutaneous nerve (*n* = 31, 32%), followed by urinary tract infection (*n* = 4, 4.2%), vascular injury needing vascular intervention (*n* = 4, 4.2%), superficial infection (*n* = 1, 1.0%) and femoral nerve injury (*n* = 1, 1.0%). In all but three of the patients with nerve injury, the symptoms resolved within 2–8 months. The four patients with vascular injury that needed vascular intervention was: three patients with occlusion of the femoral artery that were treated with endovascular thrombectomy on the same (*n* = 2) or the following (*n* = 1) day, and one patient with a perioperative injury to the femoral artery that was treated with open surgical repair during the PAO operation. These vascular events occurred scattered from 2012 to 2022.

## Discussion

The main finding of this study was that about one fifth (22%) of the patients were subsequently operated with THA, and the median time to this procedure was close to 5 years (57 months).

Our numbers on conversion to THA were in line with a study by Willey et al. including 167 hips performed by a single surgeon and a minimum 10-year follow-up, where they defined “composite failure” as conversion to THA or modified Harris Hip Score below 70 [10]. They reported a failure rate of 43%, whereof 20% THA. In contrast, our number of conversion rate to THA was substantially higher than the 6% reported in a systematic review and meta-analysis by Tan [6]. This difference might be explained by their shorter follow-up time; mean 54 months compared to our median 99 months. Accordingly, they reported that longer follow-up time (beyond 6 years) was a negative prognostic factor. Interestingly, they reported that their mean time to secondary THA was 4.7 years, which was actually longer than the follow-up time for the whole cohort, and a number close to ours. This somewhat notable result was most likely caused by the wide range (1-336 months) in their follow-up time, an expected consequence of the systematic review and meta-analysis design of the study. In a later meta-analysis by Ahmad et al., the 5-year survival (regarding THA) after PAO was 96%, and the 10-year survival was 91% [18]. Although encouraging numbers, no results were presented regarding the time to the secondary THA or the actual number of patients that were eligible to be included in the survival analysis at the different time points, making their results somewhat difficult to interpret and compare. In a recent single center study by West et al., they reported a somewhat longer time (8 years versus our 5 years) to secondary THA after PAO in a retrospective series of 103 hips [19]. Their mean age at the time of THA surgery was similar to our patients (40 versus 43 years). Moreover, they reported excellent implant survival and good clinical outcomes, concluding that prior PAO does not seem to compromise outcomes in secondary THA. However, contradictive results were reported in a previous systematic review by Shapira et al., who concluded that PAO may entail challenges on subsequent THA surgery, illustrated by higher intraoperative blood loss, lower consistency in cup positioning and compromised patients reported outcomes [20]. All the above presented results highlight the need for long follow-up times if THA surgery is to be used as an outcome. Although not very recently published, Lerch et al. reported 2016 in an impressive long-term follow up that the survivorship was approximately 30% after 30 years in their first 75 patients [21]. One theoretical possible reason for our “high” conversion rate compared to other studies could be that our results were actually due to a “learning curve”. However, PAO surgery has been performed at our institution since 1994, and the surgery was from the beginning and until now always done by at least 2 experienced surgeons together. Early results (1994–2002) from our institution have been published previously [22].

We found that high age (≥ 30 years) and preoperative osteoarthritis (Tönnis grade 2) were associated with an increased rate of secondary THA surgery. These findings were in line with the study by Willey et al. who also reported that increasing age and preoperative OA were both risk factors for failure [10]. Although the association with osteoarthritis in our series must be considered as weak (only 2 patients, and a hazard ratio confidence interval of 2.6–384 in multivariable analysis), these findings were not surprising, and these factors are probably considered as relative contraindications for PAO surgery in many institutions. As an example, the inclusion criteria for the meta-analysis by Ahmad et al. stated that only patients aged < 40 years and with Tönnis grade < 3 were included [18]. In contrast, Fischer et al. included only patients > 30 years, and reported significant postoperative pain and improved joint functionality in 48 patients followed for 2 years [12]. They suggested that even patients over 40 years can benefit from PAO surgery. However, choosing between PAO and THA in young and active patients with hip dysplasia is challenging. In a systematic review and meta-analysis on this topic by Kim and Kim, they reported that the incidences of postoperative complications and revision surgery were not different between THA and PAO groups [23]. However, postoperative pain was less in the THA group, and the activity score was higher in the PAO group. Somewhat contradictive results were reported by Parilla et al. in a retrospective cohort study comparing patients < 40 years after PAO due to dysplasia or THA due to secondary osteoarthritis [24]. The patients were followed for around 10 years, and they found no differences in complications, reoperations or functional scores between the groups. One could still argue that a PAO operation is joint-preserving, and can be followed by a THA if necessary at a later stage in life, whereas an arthroplasty is an end-stage procedure when it comes to the hip joint. Especially aseptic loosening has been a major concern for young patients after THA, and therefore a delay in arthroplasty surgery could be beneficial for the individual patient [25]. In the process of decision making, it is of utmost importance that the individual patient is well informed about specific risks associated with each of the options, for example periprosthetic fractures and dislocations in the case of THA, and osteoarthritis and non-union in the case of PAO.

Smoking was identified as a predictor for THA surgery in the regression analyses. Although the total number of smokers was low (4 patients), it must be considered as an important finding confirming smoking as an independent risk factor for orthopaedic surgery in general, and especially bone healing [26]. As this is a modifiable risk factor, it is probably mandatory in many institutions that PAO patients stop smoking prior to surgery.

A total of 6 patients had an LCEA of > 25° on preoperative radiographs. Although the LCEA is commonly used for screening, it can be difficult to measure and the exact value for individual patients must be interpreted with some caution [27]. We could not demonstrate any associations between acetabular radiological outcomes (AWI, PWI or ARI) and the risk for THA in our analyses. In the series by Stetzelberger et al., they demonstrated a correlation between a deficient AWI and the risk for conversion to THA, but failed to do so with the PWI [28]. Despite this, we still think that positioning of the osteotomy is of great importance and a crucial part of the operation. Especially as this a factor that can be actively influenced by the surgeon.

We found that 10 patients were reoperated due to other unexpected reasons than subsequent THA. In addition, a large number of patients (*n* = 39) were reoperated with extraction of implants/screws, which might be considered as a minor or even expected event. Taken together, these numbers are probably acceptable with regards to the type of complex surgery that a PAO operation constitutes. The same was for the other adverse events which affected 43% of the patients, and where the by far most common one was a transient nerve injury of the lateral femoral cutaneous nerve. Still, we had four patients with vascular injuries that required intervention. These injuries were most likely caused by poor retractor and/or chisel/saw placement when performing the osteotomy of the superior ramus. A slightly less invasive surgical approach was reported 2008 by Troelsen et al. [29], and this might decrease the risk for some of the adverse events that we had in our cohort. Although there is no universal standard on how to report reoperations and adverse events making comparisons between studies difficult, our numbers seem to be in the same magnitude as many other studies [6].

### Strengths and limitations

A major strength of this study was the large number of included patients. Another strength was the relatively long follow-up time, allowing for the capture of late as well as early reoperations and adverse events. Furthermore, the extraction of data from the national Swedish Arthroplasty Register using the unique Swedish personal identification number ensured that data on the secondary THA operations was collected with high reliability. There were several limitations with the study, and its retrospective design being an obvious one. In addition, although 8 years follow-up time is relatively long in comparison to most other studies, it could still be of great interest with longer follow-ups as an increasing number of patients could be expected to suffer from complications and subsequent THA operations over time. Another limitation was the possibility that some adverse events could have been missed if they were treated at other hospitals. However, since the Karolinska University hospital is the only hospital in the region treating these patients, the likelihood for this remains limited. Another limitation was the lack of standardized follow-ups with functional outcomes. Berwin et al. reported high rate of long-term patient satisfaction in several PROMs in 90 PAO patients after 6.5 to 20 years [11]. In addition, Willey et al. reported higher levels of physical function, lower pain severity and less hip impairment in PROMs [10].

Finally, this was a consecutive series of patients (except 2 patients who preferred to opt-out). The consecutive design could be both a strength, with no selection of patients, and a limitation as it means that also outlier patients are included, who might skew the overall results. However, we think that our results are highly generalizable and represents a typical large university hospital clinic.

## Conclusion

In this eight-year follow-up of 96 consecutive patients the rate of conversion to THA was about one fifth, and in these cases the time to the THA surgery was around five years. Taken together with the relatively low number of serious complications, the PAO procedure can be a suitable alternative in young patients with symptomatic dysplasia of the hip in order to avoid, or at least delay, hip arthroplasty.

## Data Availability

The datasets used and/or analysed during the current study are available from the corresponding author on reasonable request.

## Abbreviations

CI: Confidence interval
HR: Hazard ratio
LCEA: Lateral center-edge angle
PAO: Periacetabular osteotomy
THA: Total hip arthroplasty
